# Perceived risk of watery diarrhea and dysentery and intended compliance with chemoprophylaxis among a deployed military population

**DOI:** 10.1186/s40794-015-0009-2

**Published:** 2015-08-19

**Authors:** Chad K. Porter, Kristen Felicione, David R. Tribble, Adam W. Armstrong, Manal Mostafa, Mark S. Riddle

**Affiliations:** 1 Enteric Diseases Department, Naval Medical Research Center, Silver Spring, MD USA; 2 George Washington University, School of Public Health and Health Services, Washington, District of Colombia USA; 3 Infectious Diseases Clinical Research Program, Uniformed Services University, Bethesda, MD USA; 4 United States Naval Medical Research Unit No.6, Lima, Peru; 5 United States Naval Medical Research Unit No. 3, Cairo, Egypt

## Abstract

**Background:**

Infectious diseases are a leading cause of morbidity among travelers to resource-limited regions and primary prevention is a cornerstone to risk reduction. Chemoprophylaxis has been successfully utilized for specific diseases.

**Methods:**

We assessed self-reported compliance to daily chemoprophylaxis among deployed US military personnel. A 21 item self-completed questionnaire was completed by military personnel during mid-deployment.

**Results:**

The perception of high disease risk was associated with an increased likelihood of compliance with daily chemoprophylaxis. However, 60 % of respondents stated they would not comply with a daily regimen.

**Conclusions:**

These data highlight the complexity of perceived risk and the difficulties with prophylactic interventions.

## Background

Infectious diseases have been and remain a leading cause of morbidity in travelers, including the deployed military. In military populations, disease prevention has emphasized preparation, education, personal protective measures, vaccines, and chemoprophylaxis [1]. The effectiveness of these measures is often dependent on the disease in question, though understanding the perceptions and attitudes towards primary prevention strategies may enable the development of more targeted interventions with higher success rates.

Vaccination has a clear history of reducing disease risk for many diseases of military importance; however, for several (malaria, travelers’ diarrhea, dengue fever, skin infections, etc.) vaccines remain unavailable. In their absence, increased use of personal protection, vector control, and chemoprophylaxis has reduced malaria incidence in endemic regions [2]. In contrast, chemoprophylaxis is not currently recommended for travelers’ diarrhea (TD), and utilization of standard public health practices to minimize disease risks has yielded little impact on TD incidence, with average monthly attack rates of 30 % among deployed populations over the past two decades [3, 4]. In addition to the burden of acute illness, evidence is increasing on the importance of several sequelae of TD [5]. This, coupled with the development of new non-absorbable antibiotics, may modify the paradigm for chemoprophylaxis [6]. In fact, such a study was recently conducted in active duty military personnel deployed to Incirlik Air Base [7]. While the data from that study highlight the potential efficacy of a specific chemoprophylactic regimen in certain regions, it is unclear whether comparable effectiveness results would be borne out due to potential issues with non-compliance. As such, we sought to assess factors associated with intended compliance to a daily regimen of chemoprophylaxis for TD as well as malaria (for comparison).

## Methods

This was a cross-sectional study utilizing a convenience sample of active duty US military personnel deployed to Iraq, Afghanistan, and the surrounding region during an in-processing brief as part of rest and recuperation (R&R) at Camp As Sayliyah in Doha, Qatar as previously described [4]. From April 2006 through September 2007, a single page, 21 item, self-completed questionnaire soliciting information on demographics, prior deployments, perceived disease risk and likelihood of compliance to a daily prophylactic was distributed to military personnel. The questionnaires were voluntary and anonymous and were designed to assess potential future compliance with a hypothetical travelers’ diarrhea prophylactic compared to theoretical compliance with malaria prophylaxis. Data were entered into Microsoft Excel (Microsoft Inc., Redmond, WA) and verified by Naval Medical Research Unit No. 3 (NAMRU-3) (Cairo, Egypt) staff.

Descriptive analyses were performed using Student’s *t* test for continuous variables and Pearson Chi-Square tests for categorical variables. Perceived disease risk was assessed by the question “What is your perceived risk of acquiring Disease X”. Logistic regression models were developed using backwards elimination to identify factors associated with an increased likelihood of compliance to daily chemoprophylaxis for TD as well as malaria. All variables with a p < 0.25 were retained in the final model. Statistical significance was set at an alpha = 0.05 and all analyses were conducted using SAS v9.3 (Cary, NC).

### Human subjects protection

This study was approved by the institutional review board at the NAMRU-3, Cairo, Egypt.

## Results

A total of 2235 questionnaires were distributed, completed and returned. Subjects were predominantly male (84.2 %), in the Army (76.3 %) and enlisted (87.3 %). The mean age of responders was 28.9 years. The majority of respondents (71.8 %) were deployed in support of Operation Iraqi Freedom and were comprised of active duty (62.9 %), reserve (18.5 %) and National Guard (17.1 %) personnel. These demographic and populations characteristics were representative of the deployment population to the region.

A significant proportion of subjects identified themselves as being at no risk for dysentery (46.7), watery diarrhea (45.8), or malaria (48.6) regardless of the country to which the subject was predominately deployed, while approximately 30 % did not know their risk for these infectious diseases (Table 1). Perceived disease risk varied by country of deployment with the highest rates of ‘high risk’ observed in individuals deployed to Afghanistan. The majority of subjects reported they would not take a daily chemoprophylactic to prevent diarrhea (61.5 %) or malaria (62.2 %) though subjects were most likely (always or very likely) to comply with daily chemoprophylaxis against malaria (17.1 %) rather than for diarrhea (10.9 %) (data not shown).

**Table 1:** Reported perceived disease risk among responders

Country predominately deployed	Disease	Perceived Risk, n(%)			
		High risk	Low risk	No risk	Don’t know/missing
Afghanistan	Malaria	28 (9.7)	100 (34.7)	96 (33.3)	64 (22.2)
	Dysentery	19 (6.6)	91 (31.6)	101 (35.1)	77 (26.7)
	Watery diarrhea	26 (9.0)	81 (28.1)	100 (34.7)	81 (28.1)
Iraq	Malaria	11 (0.7)	294 (18.6)	801 (50.7)	473 (30.0)
	Dysentery	35 (2.2)	300 (19.0)	773 (49.0)	471 (29.8)
	Watery diarrhea	51 (3.2)	292 (18.5)	759 (48.5)	477 (30.2)
Other/Unknown	Malaria	4 (1.1)	78 (21.2)	189 (51.4)	97 (26.4)
	Dysentery	5 (1.4)	84 (22.8)	170 (46.2)	109 (29.6)
	Watery diarrhea	12 (3.3)	91 (24.7)	164 (44.6)	101 (27.5)

After controlling for covariates, a high perceived risk of diarrhea and malaria was associated with a significant increase in the likelihood of compliance with daily chemoprophylaxis compliance (OR: 3.8 and 4.9, respectively) (Table 2). Additionally, military officers were more likely to comply with daily chemoprophylaxis for diarrhea and malaria compared to enlisted respondents. Similarly, those in the Navy and Air Force were more likely to comply with chemoprophylaxis against malaria compared to those in the Army.

**Table 2:** Multivariate analyses of factors associated with the likelihood of compliance to a once-a-day pill for prevention of diarrhea or malaria

Significant Risk Factors	OR (95 % CI) of increased likelihood of compliance with daily chemoprophylaxis	
	Diarrhea	Malaria
Perceived High Risk	3.78 (2.44–5.92)	4.88 (2.52–9.46)
Officer rank	1.61 (1.09–2.37)	2.21 (1.59–3.06)
Branch	--	
Army		1.00 (ref)
Air Force		2.79 (1.55–5.03)
Marines		0.64 (0.40–1.03)
Navy		1.62 (1.11–2.37)

Hosmer and Lemeshow Goodness-of-fit chi-square of 0.31 and 1.43 for diarrhea and malaria models, respectively

## Discussion

The majority of respondents indicated no perceived risk to the diseases of interest including dysentery or watery diarrhea. Recent studies have estimated travelers’ diarrhea rates among deployed military personnel to be approximately 30 cases per 100 person-months [3]. Because of the consistent and fairly high prevalence of TD among similar populations, the low proportion perceiving watery diarrhea and dysentery as “high risk” (less than 5 %) is discordant with anticipated responses, and it may be possible the term “risk” was perceived to mean something other than the potential for incident illness. This is supported by the minimal difference in perceived high risk of watery diarrhea and malaria; a disease with a much lower incidence rate; though it is notable that malaria risks in the two theaters of operation is different [4].

In addition to low rates of perceived high disease risk, a high proportion of respondents reported they would never take a daily pill to prevent diarrhea or malaria. For malaria, these estimates are consistent with historical reports of low chemoprophylaxis compliance; however, targeted intervention efforts have been shown to increase compliance rates and this is why directly observed therapy has been put into practice in the Department of Defense [8]. Chemoprophylaxis is not currently recommended for TD, though the development of new non-absorbable antibiotics and risk of post-infectious sequelae may modify the paradigm [9]. Nonetheless, given the challenges associated with behavioral modification, a major emphasis on prevention should continue to be vaccination when feasible.

It is unclear if increased perceived disease risk would meaningfully modify the likelihood of taking daily chemoprophylaxis. For example, a population of vacationers traveling for a significantly shorter period of time compared to active duty military members may perceive greater disease risk, thus resulting in increased compliance of daily chemoprophylaxis. Additionally, it is unclear if increased knowledge regarding the relationships between illness prevention, infection prevention, and theoretical sequelae prevention would alter attitudes toward daily chemoprophylaxis. Furthermore, the reasons for variability in compliance across military-specific characteristics are unclear highlight an area for future study. If prevention of TD is shown in future studies to decrease sequalae, issues of long-term daily chemoprophylaxis compliance could be explored in deployment settings.

## Conclusions

Given the overall low perceived risk of diarrhea and the strong association between a high perceived disease risk and compliance, daily chemoprophylaxis may be a sub-optimal means of disease prevention. Additional studies are needed to better explore the knowledge, attitudes and practice of US military personnel as well as other travel populations.
